# Supplementary material for: Niacin‐mediated Tace activation ameliorates CMT neuropathies with focal hypermyelination
Source: EMBO Mol Med. 2016 Oct 31;8(12):1438–54. doi: 10.15252/emmm.201606349 (PMC5167133; doi:10.15252/emmm.201606349)
Supplement: Supplementary file 1 — Expanded View Figures PDF [file EMMM-8-1438-s001.pdf]

Expanded View Figures

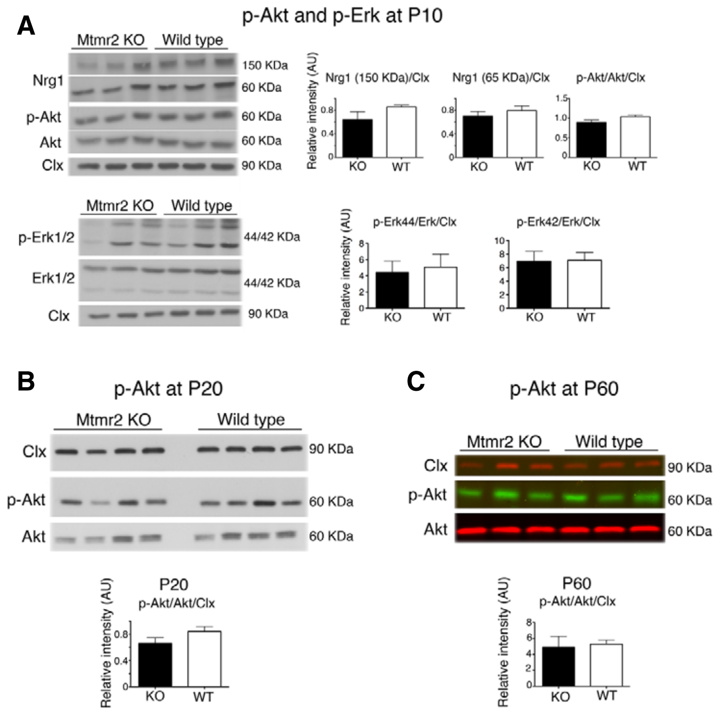

Figure EV1. Expression levels of Nrg1 and phosphorylation of Erk and Akt in *Mtmr2*$^{-/-}$ nerves.

A Phosphorylation of either Akt (S473) or Erk1/2 (44 and 42 kDa) and Nrg1 expression levels are similar between WT and *Mtmr2*$^{-/-}$ sciatic nerves at P10, with quantification. Representative of two independent experiments. Nrg1 (150 kDa), P = 0.7; Nrg1 (65 kDa), P = 0.4; p-Akt, P = 0.2; p-Erk 44, P = 0.7; p-Erk 42, P = 1, two-tailed Mann–Whitney *U*-test. Clx, calnexin.

B, C Phosphorylation of Akt (S473) at P20 (B) and at P60 (C) is similar between WT and *Mtmr2*$^{-/-}$ sciatic nerves, with quantification. Representative of two independent experiments. p-Akt, P = 0.2 at P20 and P = 0.7 at P60, two-tailed Mann–Whitney *U*-test. Clx, calnexin.

Data information: Results are expressed as mean ± SEM.
Source data are available online for this figure.

 © 2016 The Authors

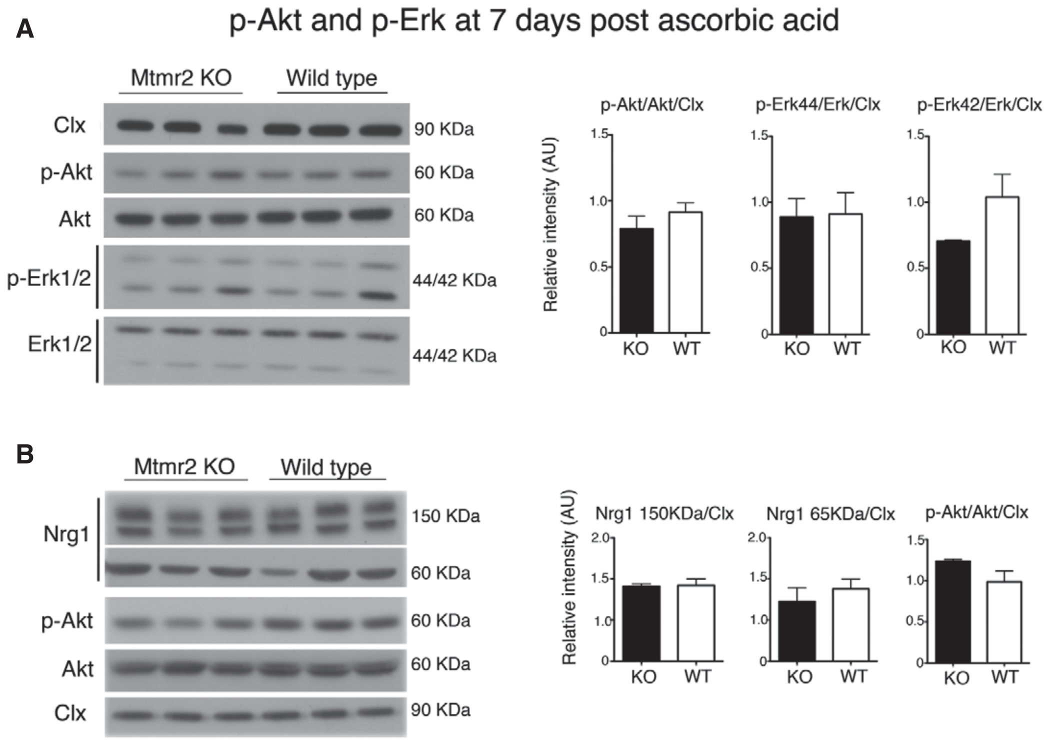

Figure EV2. Expression levels of Nrg1 and phosphorylation of Erk and Akt in *Mtmr2*$^{-/-}$ co-cultures at 7 days postdifferentiation.

A Western blot analysis and corresponding quantification of lysates from *Mtmr2*$^{-/-}$ and WT DRG explants after 7 days of ascorbic acid treatment. Each lane is a lysate from at least 10 DRGs per condition/genotype. Phosphorylation of either Akt (S473) or Erk1/2 (44 and 42 kDa) is similar between WT and *Mtmr2*$^{-/-}$ explants. Representative of two independent experiments.

B Expression levels of either Nrg1 full-length (150 kDa) or cleaved Nrg1 (65 kDa) are similar between the two genotypes. p-Akt, P = 0.353 upper panel and P = 0.1373 lower panel; p-Erk 44, P = 0.9284; p-Erk 42, P = 0.1274; Nrg1 (150 kDa), P = 0.8897; Nrg1 (65 kDa), P = 0.4966; two-tailed Mann–Whitney U-test. Clx, calnexin.

Data information: Results are expressed as mean ± SEM.
Source data are available online for this figure.

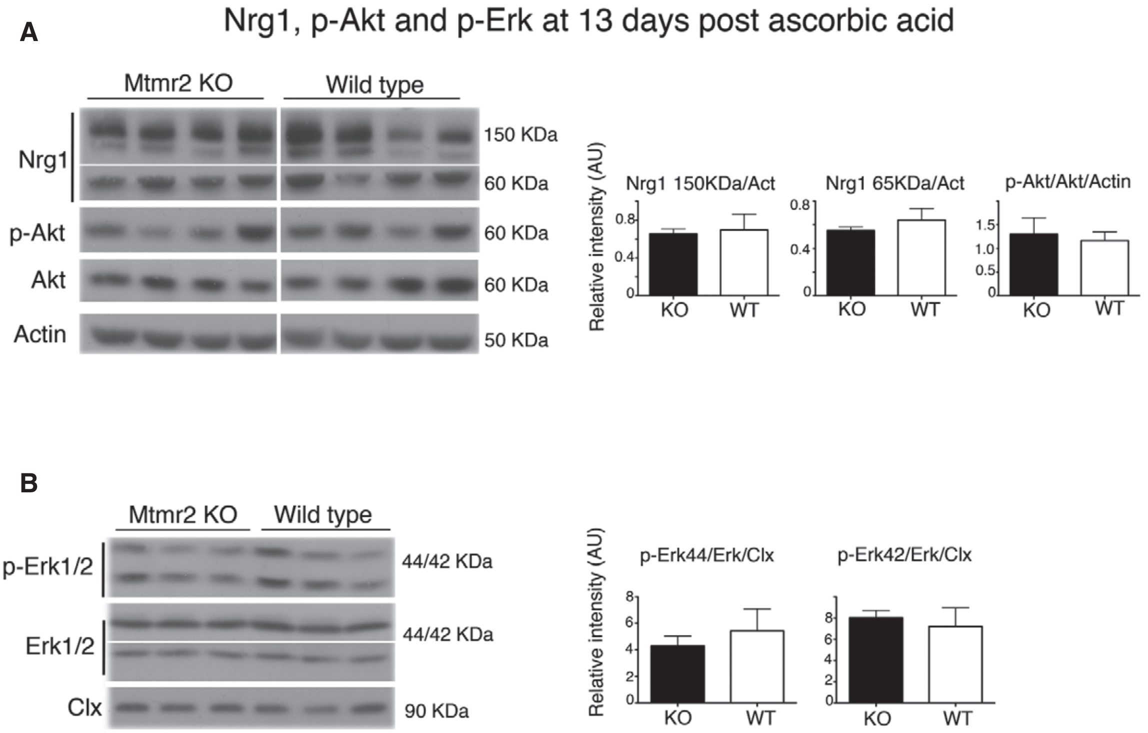

Figure EV3. Expression levels of Nrg1 and phosphorylation of Erk and Akt in *Mtmr2*$^{-/-}$ co-cultures at 13 days postdifferentiation.

A, B Western blot analysis and corresponding quantification of lysates from *Mtmr2*$^{-/-}$ and WT DRG explants after 13 days of ascorbic acid treatment. Each lane is a lysate from a pool of at least 10 DRGs per condition/genotype, n = 3/4 different pools per genotype. Results are mean ± SEM; Nrg1 (150 kDa) P = 1; Nrg1 (65 kDa) P = 0.4857; p-Akt P = 1; p-Erk 44 P = 0.7; p-Erk 42 P = 1; two-tailed Mann–Whitney U-test. Clx, calnexin.

Source data are available online for this figure.

Figure EV4. Niacin acts through Tace to modulate myelination in *Mtmr2*$^{-/-}$ cultures.

A Quantitative RT–PCR analysis using mRNA from isolated rat Schwann cells transduced with Tace shRNA LV (three different harpins tested). NI, not infected; SCR, scramble shRNA LV. Representative of two independent experiments.

B Quantification of Mbp-positive myelin segments in wild-type cultures transduced with Tace shRNA LVs as compared to NI and transduced with scramble shRNA. DRGs/coverslips: NI n = 6; SCR n = 4; sh#1 n = 4. P = 0.0181, nonparametric one-way ANOVA, followed by Dunn's *post hoc* test.

C Representative of *Mtmr2*$^{-/-}$ explants transduced with scramble shRNA (GFP tag) shows that neurons were preferentially transduced by LVs when infection was made 1 day after plating the DRGs.

D, E Representative confocal images of *Mtmr2*$^{-/-}$ co-cultures transduced using LV expressing shRNA scramble (SCR) or Tace shRNA #1 and treated or not treated (NT) using 5 mM niacin, with quantification in (E). N = 6 DRGs/coverslips per condition; 200 fibers scored per condition; P = 0.0425, nonparametric one-way ANOVA, followed by Dunn's *post hoc* test.

Data information: Results in (A, B, and E) are mean ± SEM. Dunn's *post hoc* test, *P < 0.05.

▶

 © 2016 The Authors

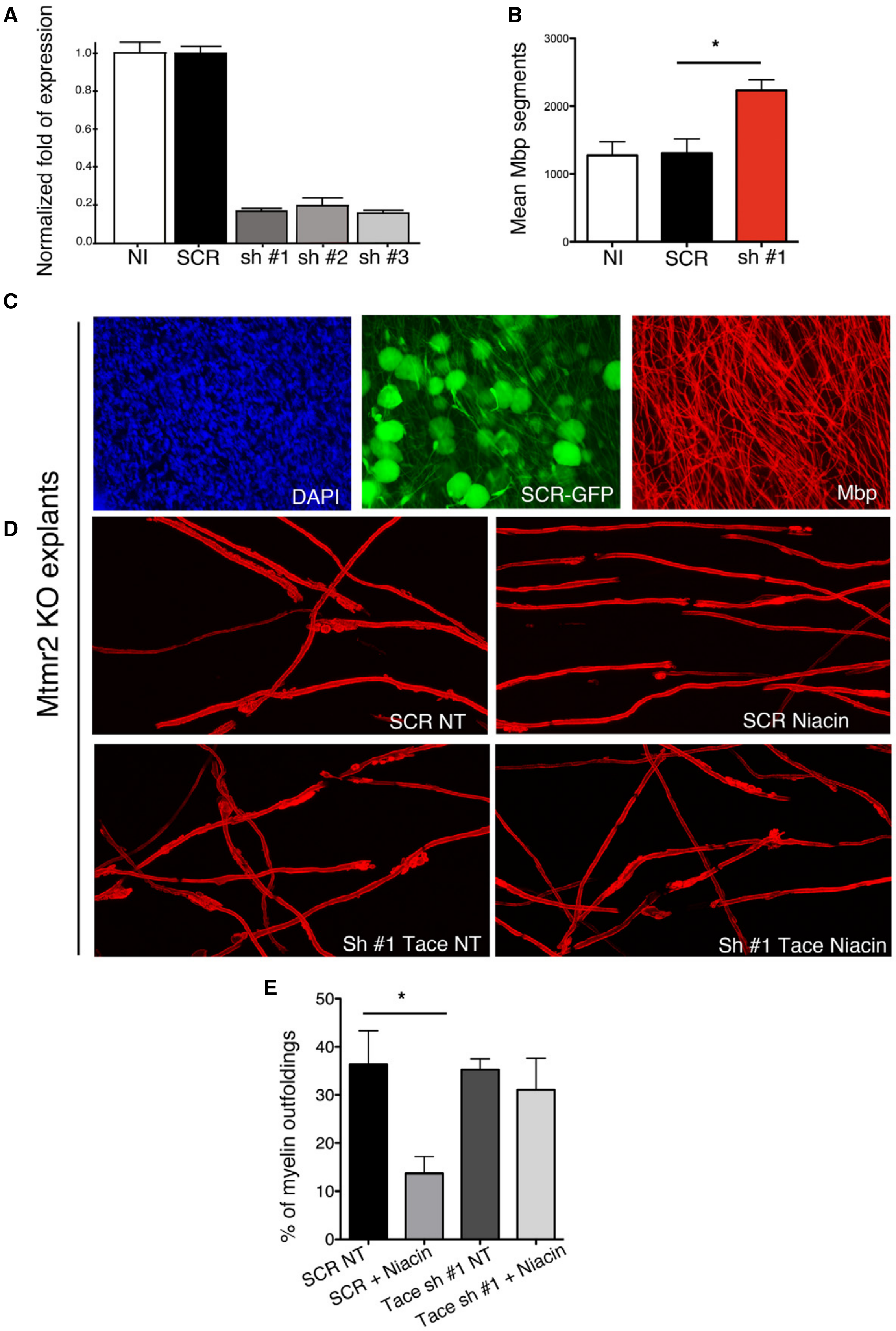
A
Normalized fold of expression
1.0
0.8
0.6
0.4
0.2
0.0
NI
SCR
sh #1
sh #2
sh #3
B
Mean Mbp segments
3000
2000
1000
0
*
NI
SCR
sh #1
C
DAPI
SCR-GFP
Mbp
D
Mtmr2 KO explants
SCR NT
SCR Niacin
Sh #1 Tace NT
Sh #1 Tace Niacin
E
% of myelin outfoldings
50
40
30
20
10
0
*
SCR NT
SCR + Niacin
Tace sh #1 NT
Tace sh #1 + Niacin

Figure EV4.

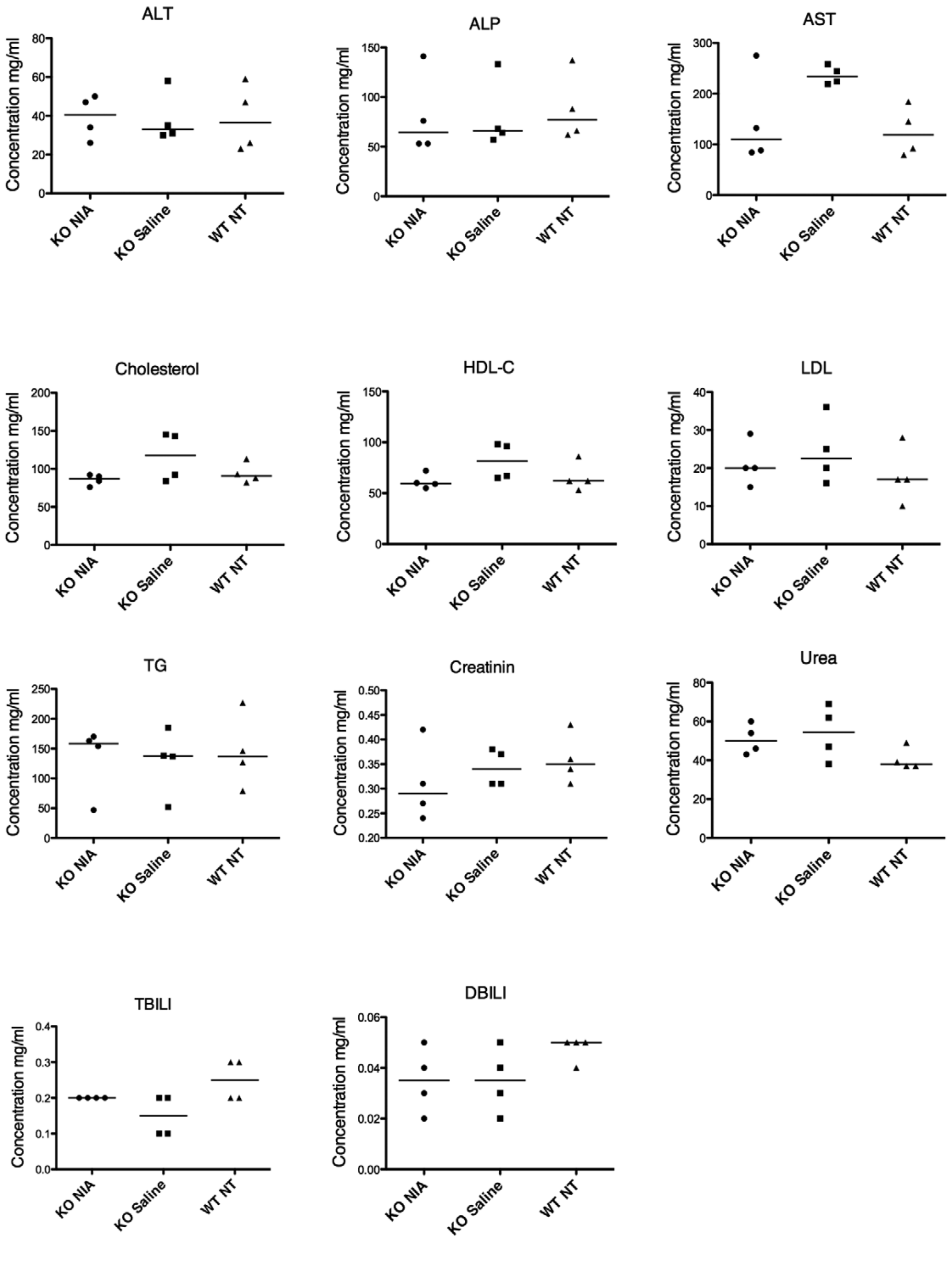

Figure EV5. Bioclinical analysis in *Mtmr2*$^{-/-}$ mice treated with Niaspan for 2 months (160 mg/kg/day) starting at P15.

ALT, alanine transaminase; ALP, alkaline phosphatase; AST, aspartate transaminase; HDL-C, high-density lipoprotein cholesterol; LDL, low-density lipoprotein cholesterol; TG, triglycerides; TBILI, bilirubin; DBILI, direct bilirubin, conjugated. Results are median values, nonparametric one-way ANOVA, followed by Dunn's *post hoc* tests. No significant differences are observed by comparing the three groups for all the tested parameters: ALT $P = 0.9436$; ALP $P = 0.7781$; AST $P = 0.1160$; cholesterol $P = 0.2895$; HDL-C $P = 0.1152$; LDL $P = 0.5451$; TG $P = 0.9260$; creatinine $P = 0.4059$; urea $P = 0.1662$; TBILI $P = 0.0639$; DBILI $P = 0.2046$, representative of two independent experiments.

 © 2016 The Authors

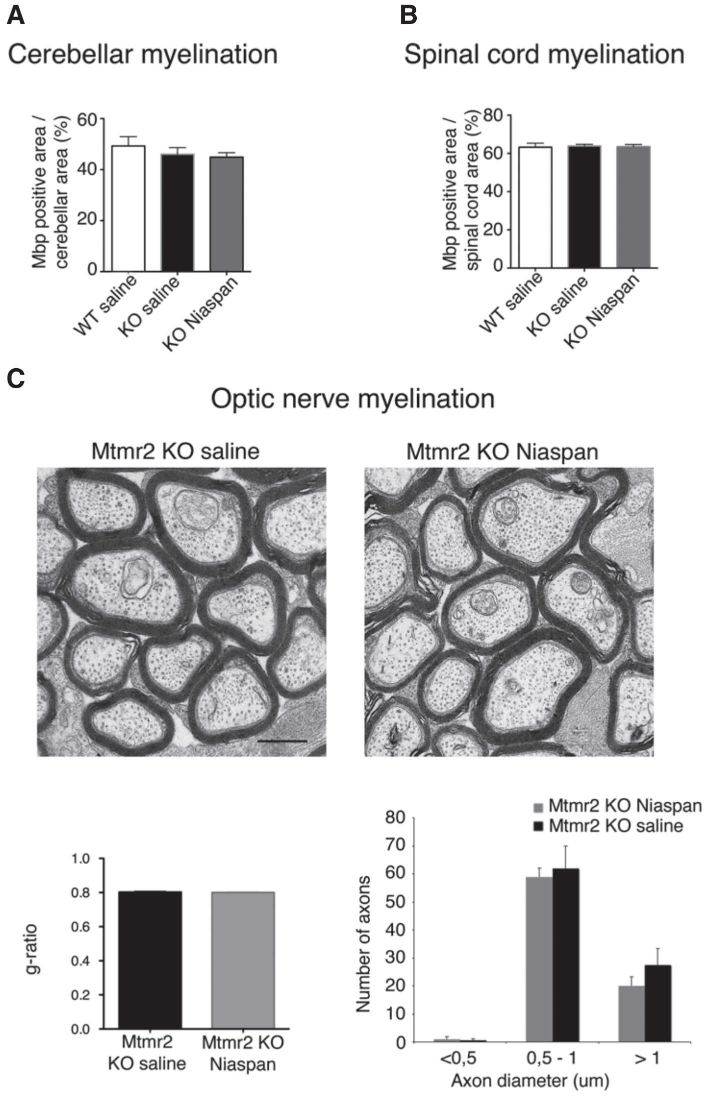

Figure EV6. Normal CNS myelination in *Mtmr2*$^{-/-}$ mice following Niaspan treatment at 2.5 months.

- A Mbp staining on frozen brain (cerebellum) sections from *Mtmr2*$^{-/-}$ mice (Niaspan- and saline-treated) and WT saline-treated mice. Quantification of Mbp staining over the area indicates that myelinated tracts are not affected by Niaspan treatment, n = 5 animals per condition, P = 0.4054, nonparametric one-way ANOVA, followed by Dunn's *post hoc* test.
- B Mbp staining in spinal cord sections (at two different levels) of *Mtmr2*$^{-/-}$ mice (Niaspan- and saline-treated) and WT saline-treated mice. Quantification of Mbp staining in the spinal cord area indicated that myelinated tracts were not affected by Niaspan treatment, n = 5 animals per condition, P = 0.9324, nonparametric one-way ANOVA, followed by Dunn's *post hoc* test.
- C Ultrastructural and g-ratio analyses of optic nerves from *Mtmr2*$^{-/-}$ saline- and Niaspan-treated mice show normal myelin thickness in the two groups of mutants, n = 5 animals per condition (*Mtmr2*$^{-/-}$ saline, 0.8 ± 0.004, 450 fibers and *Mtmr2*$^{-/-}$ Niaspan 0.8 ± 0.002, 400 fibers; n = 5 animals per genotype; P = 0.5728, repeated-measures ANOVA). Scale bar, 1 μm.

Data information: Results are expressed as mean ± SEM.

© 2016 The Authors
