# Supplementary material for: Niacin‐mediated Tace activation ameliorates CMT neuropathies with focal hypermyelination
Source: EMBO Mol Med. 2016 Oct 31;8(12):1438–54. doi: 10.15252/emmm.201606349 (PMC5167133; doi:10.15252/emmm.201606349)

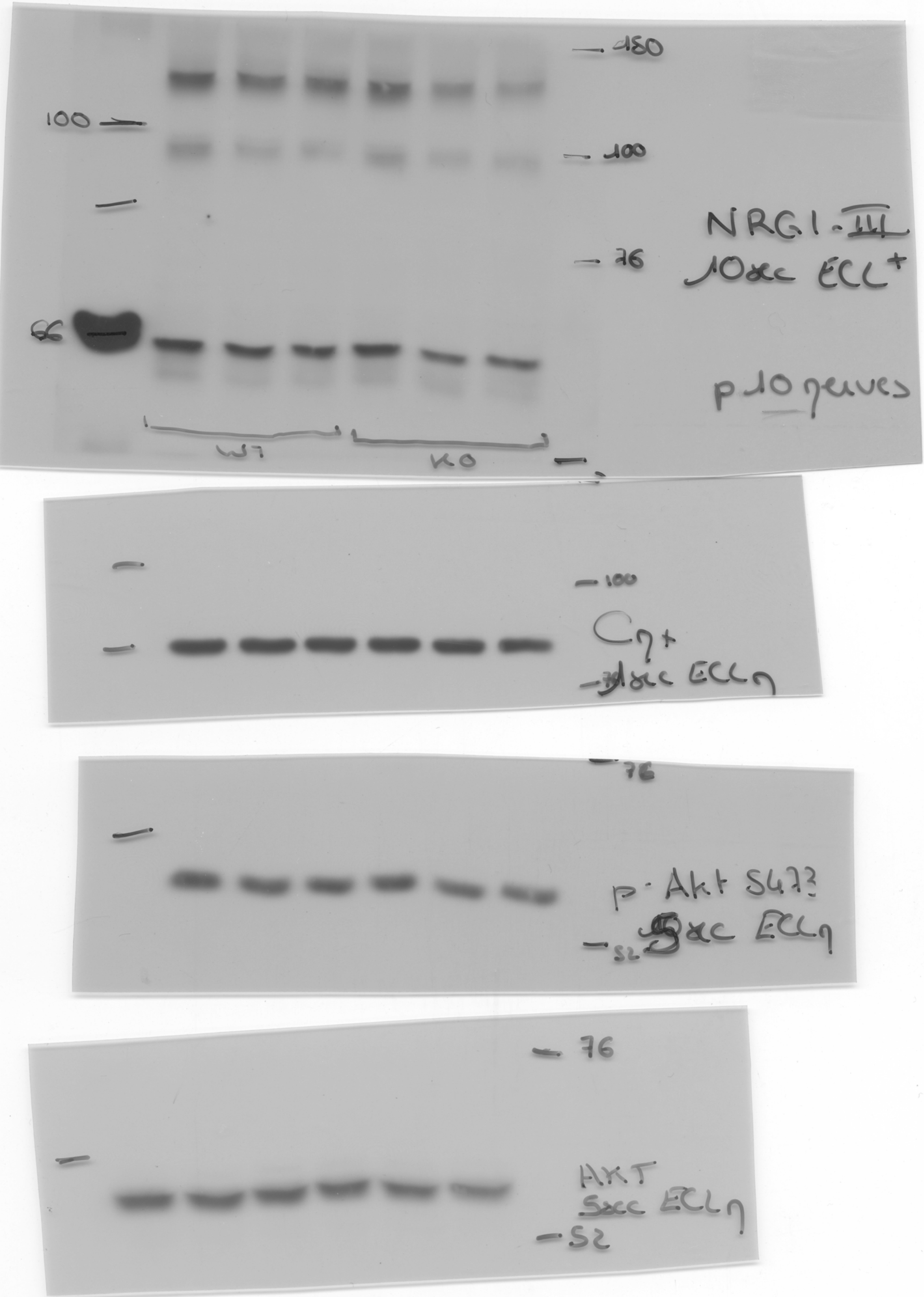
180
100
100
76
NRG1-III
10sec ECL+
66
p10 nerves
WT
KO
100
76
p-Akt S473
52
76
AKT
5sec ECLn
52

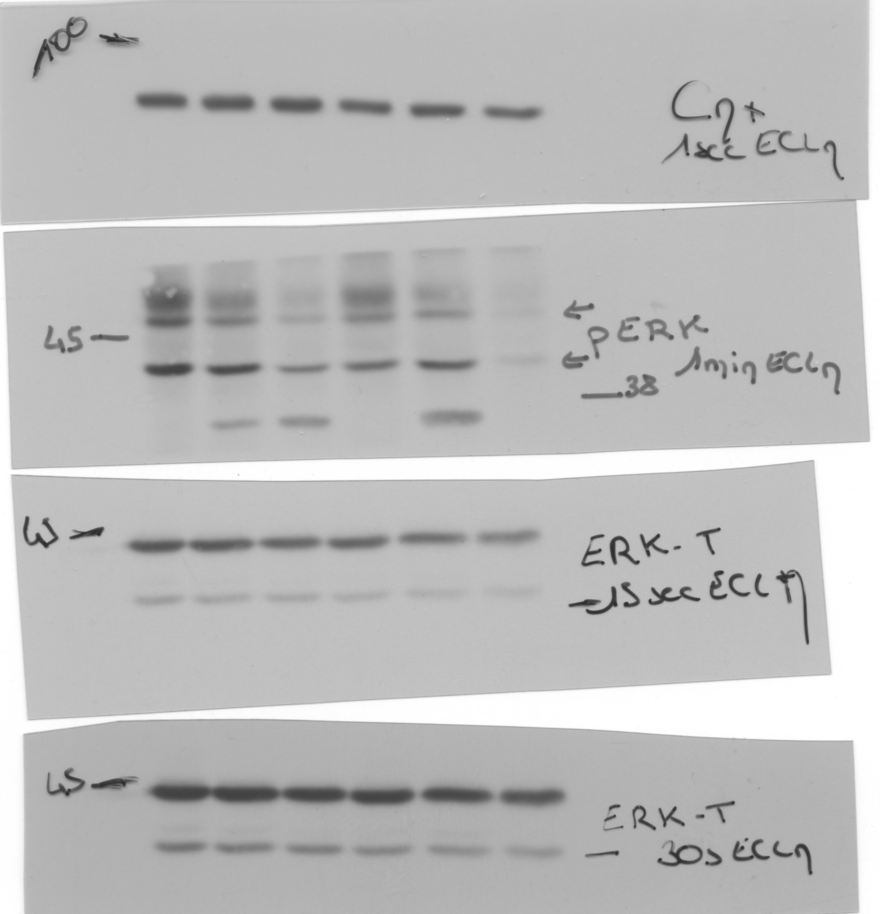
100
Cnx
1sec ECL
45
pERK
1min ECL
38
45
ERK-T
15sec ECL
45
ERK-T
30s ECL

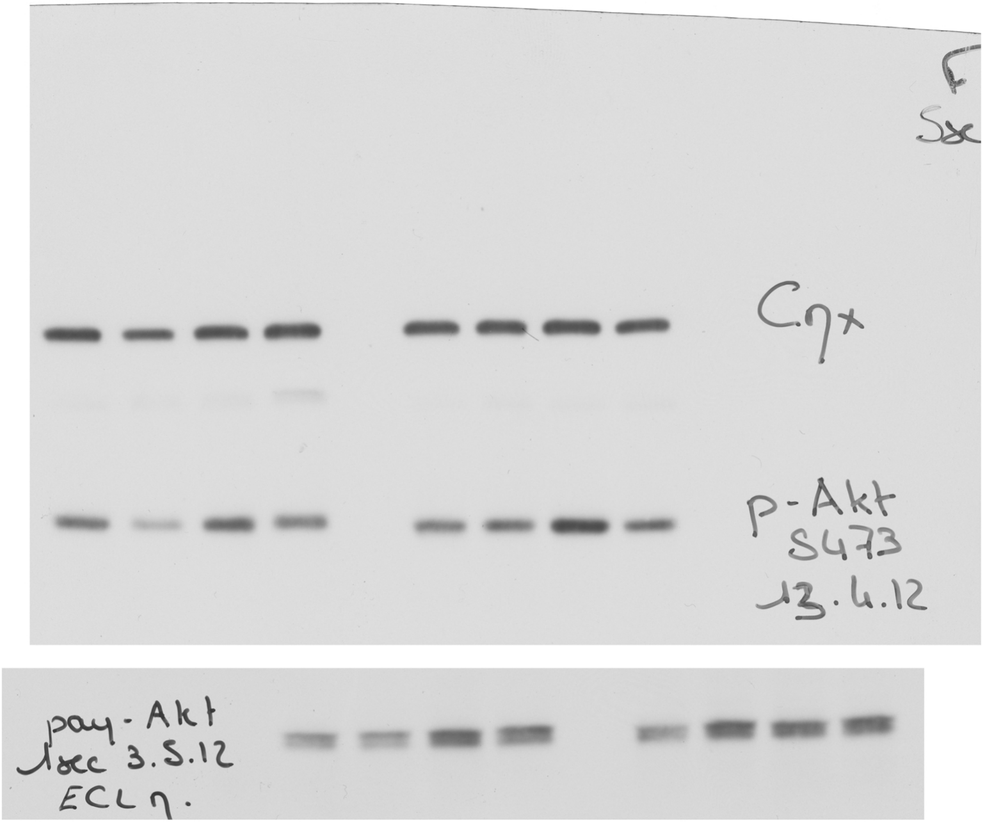

Figure EV1 panel B

Figure EV1 panel C

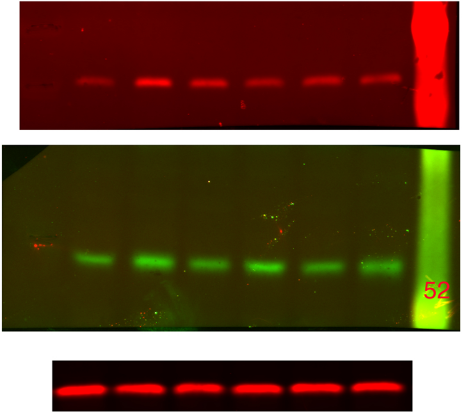

Supplement: Supplementary file 2 — Source Data for Expanded View [file EMMM-8-1438-s009.zip › EMM_06349_EV_Source_Data/EMM_06349_source_data_FigEV1.pdf]
