# Supplementary figures and images for: Niacin‐mediated Tace activation ameliorates CMT neuropathies with focal hypermyelination
Source: EMBO Mol Med. 2016 Oct 31;8(12):1438–54. doi: 10.15252/emmm.201606349 (PMC5167133; doi:10.15252/emmm.201606349)

Figure EV2 panel A

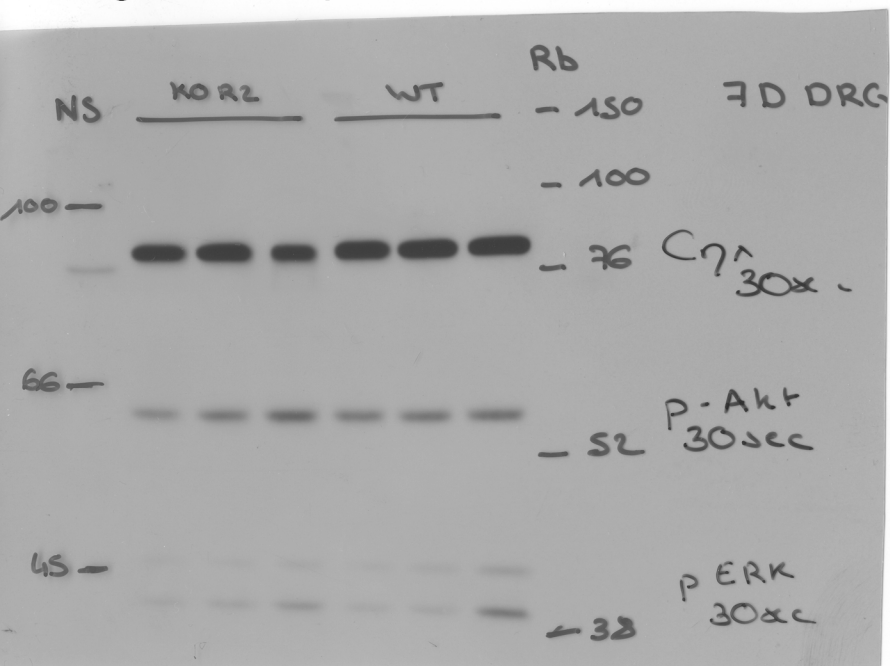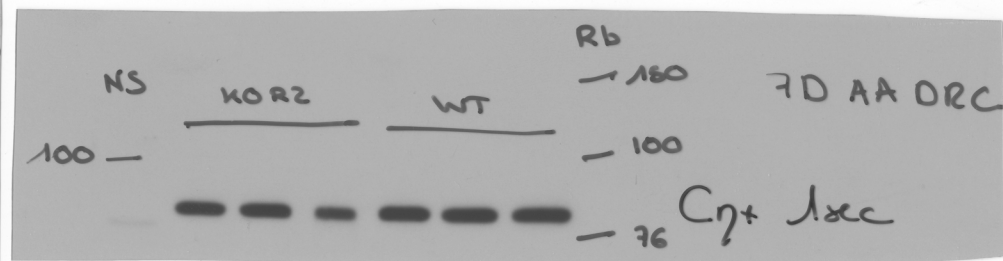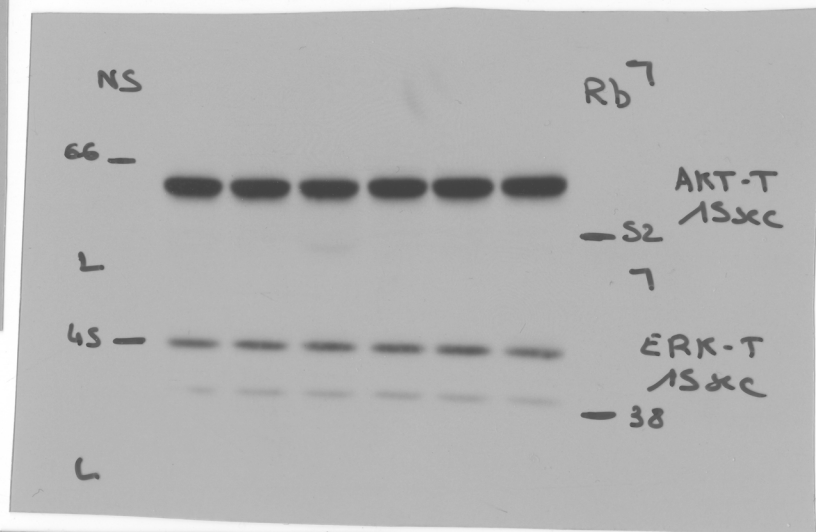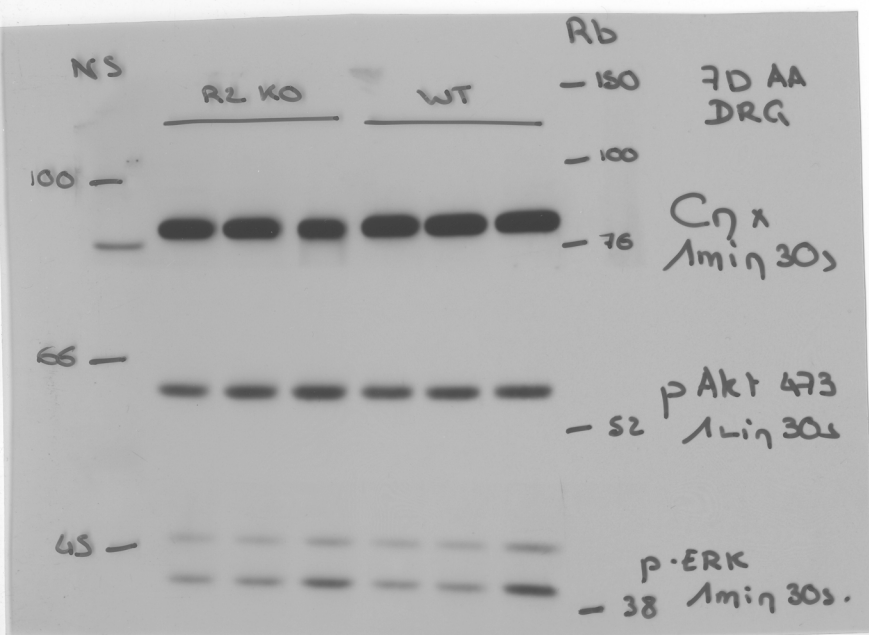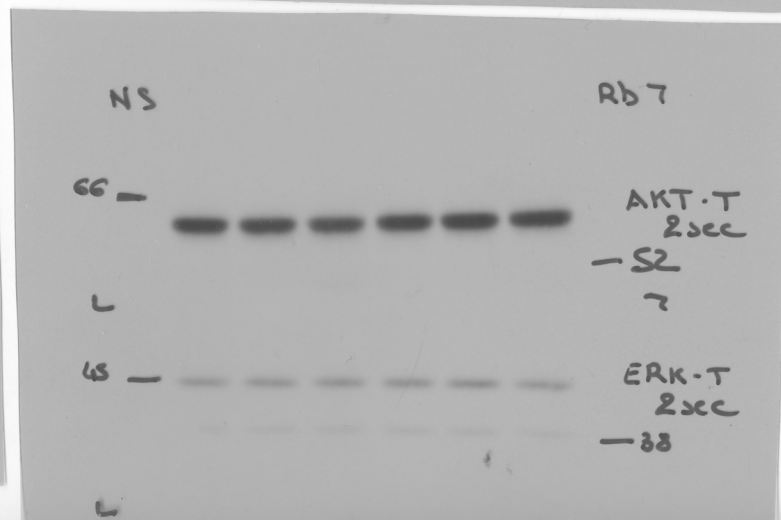

Figure EV2 panel B

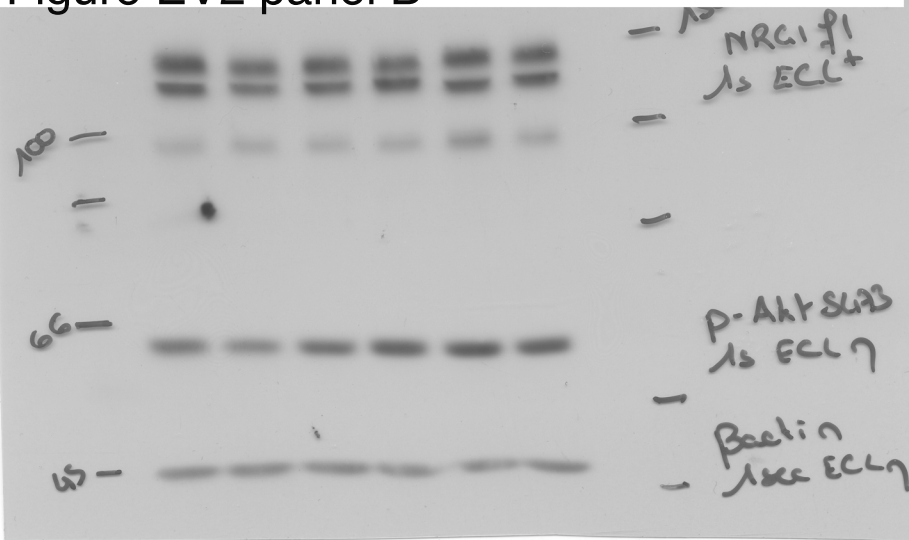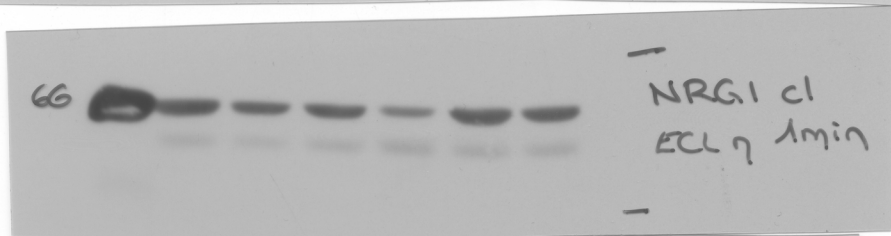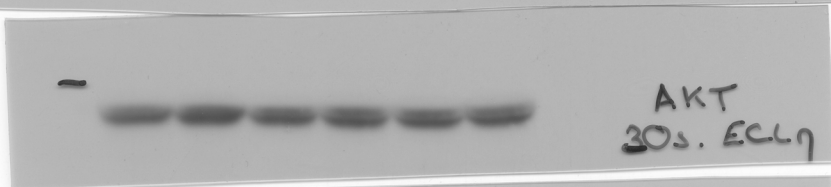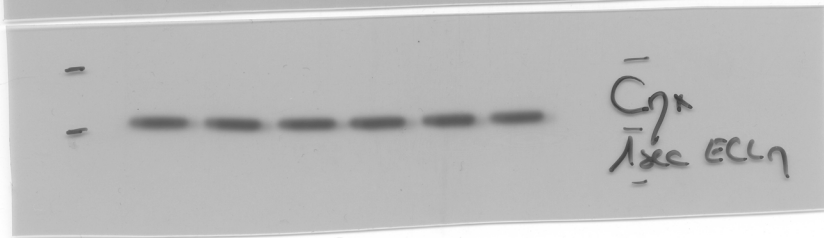

Supplement: Supplementary file 2 — Source Data for Expanded View [file EMMM-8-1438-s009.zip › EMM_06349_EV_Source_Data/EMM_06349_source_data_FigEV2.pdf]

Figure EV3 panel A

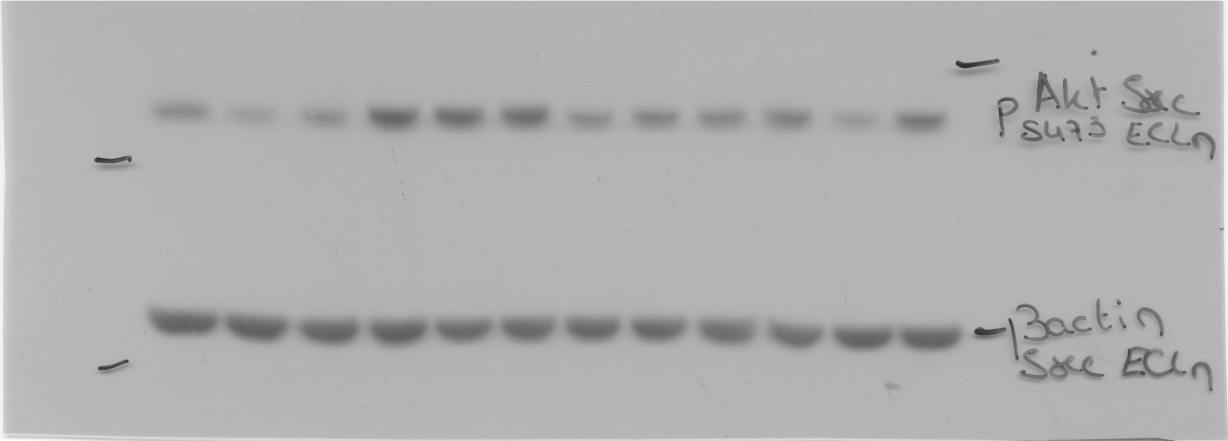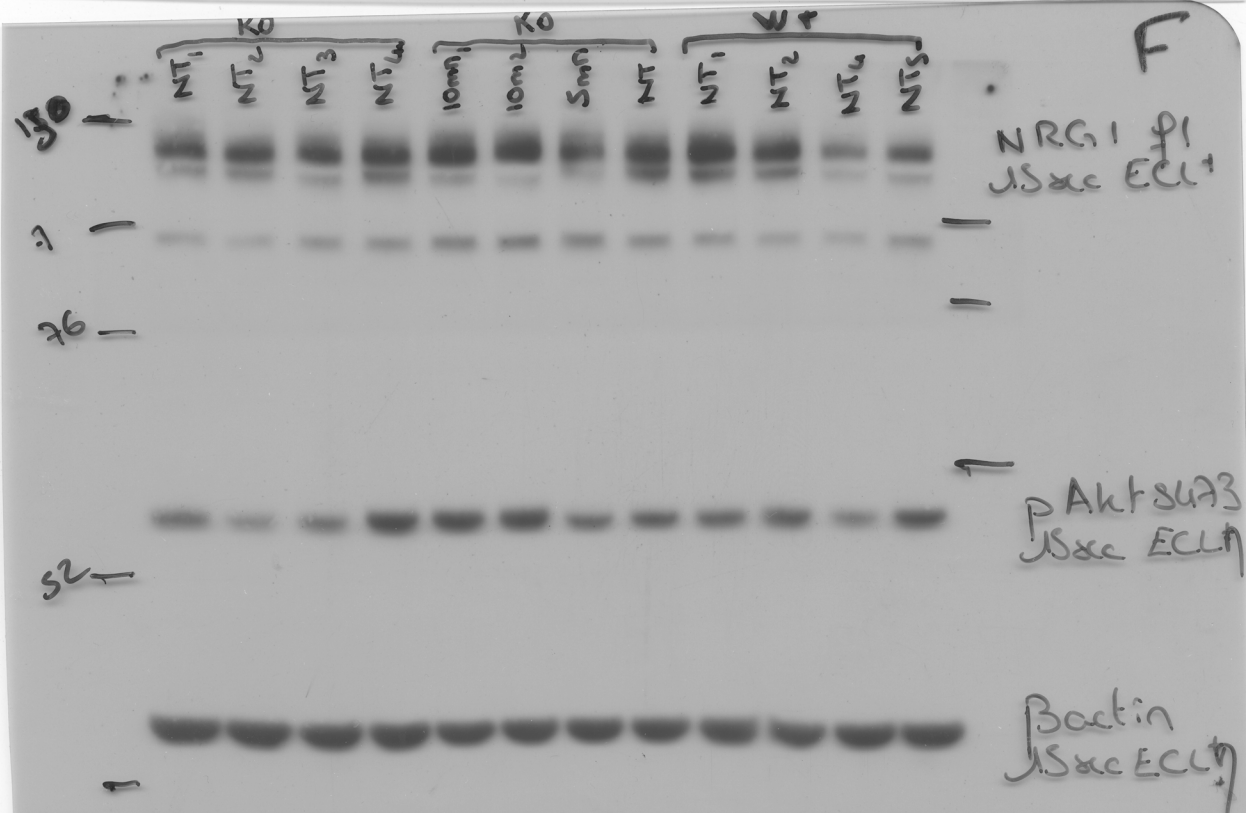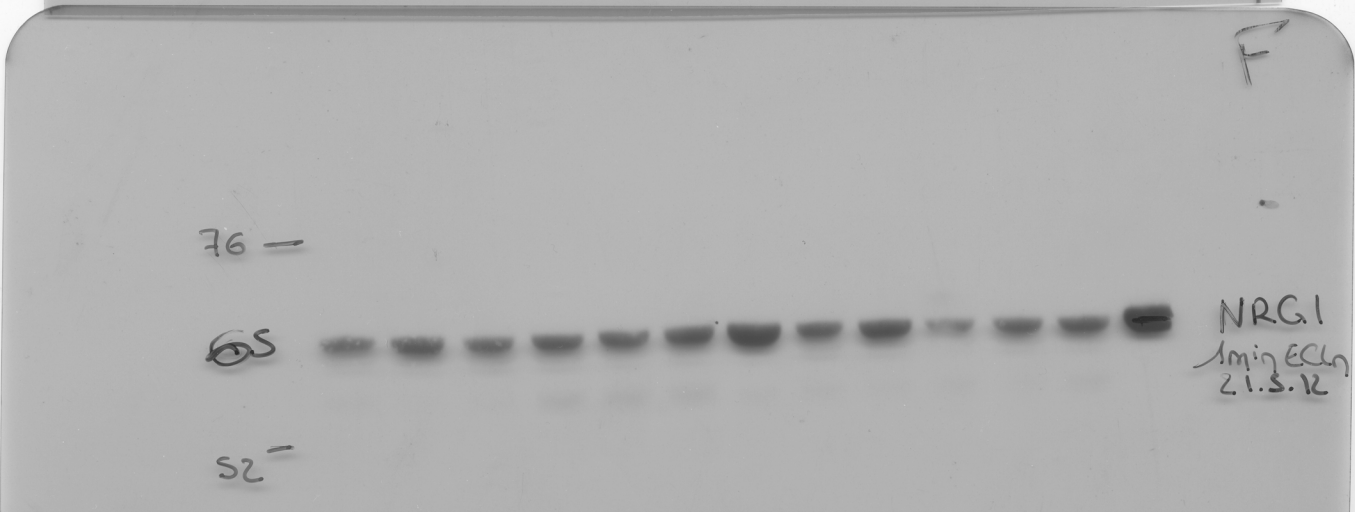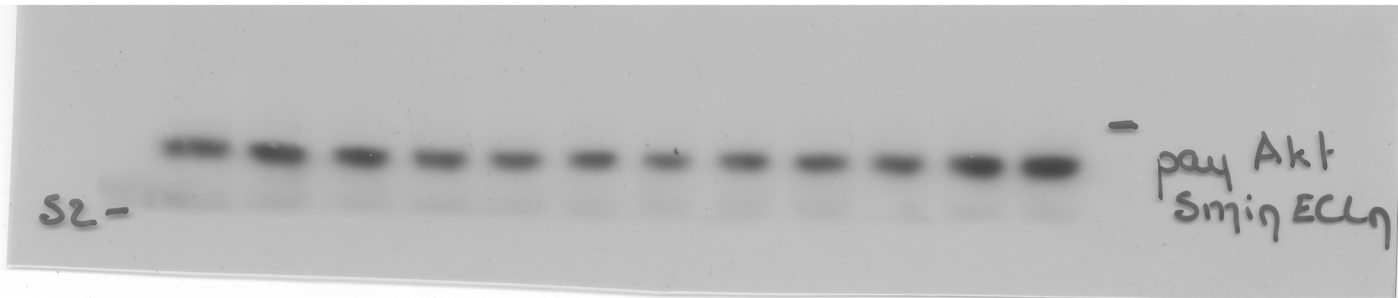

Figure EV3 panel B

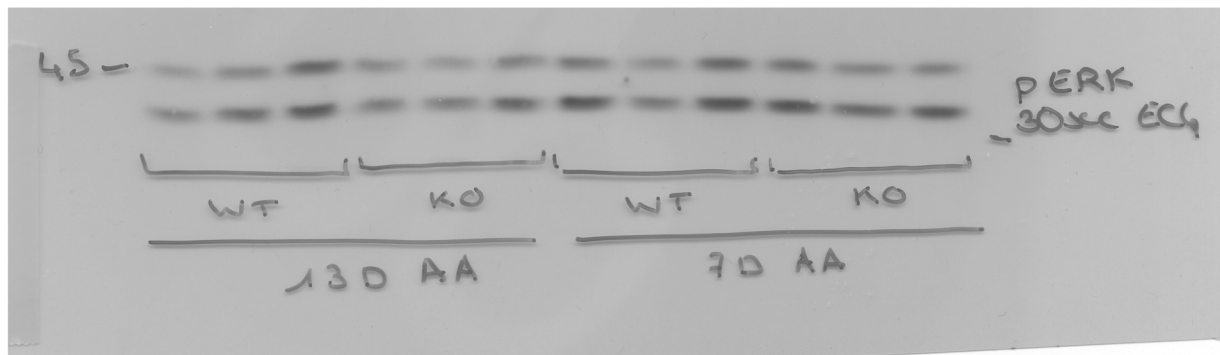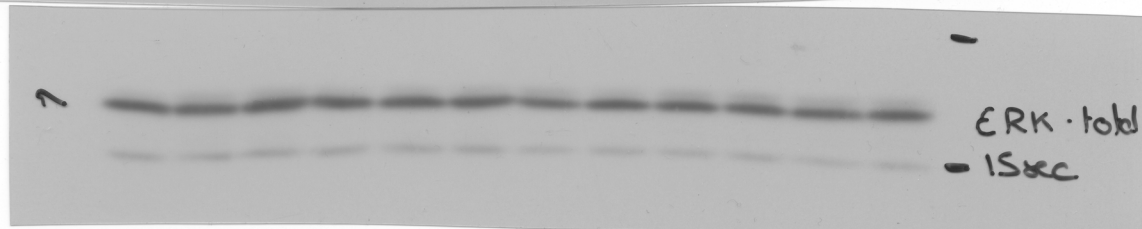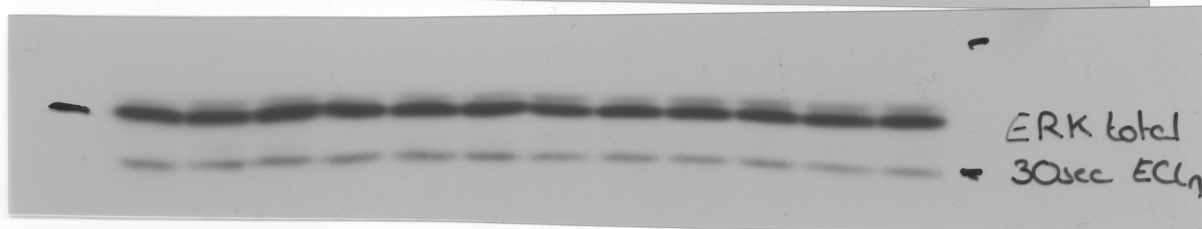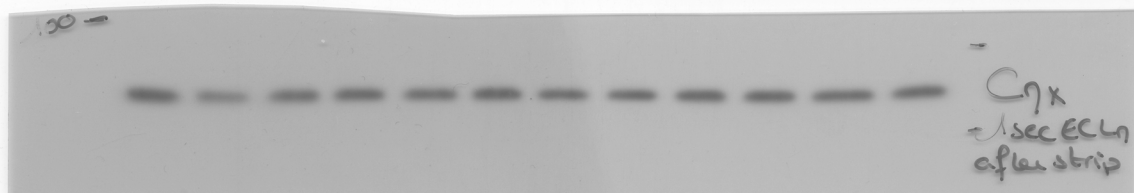

Supplement: Supplementary file 2 — Source Data for Expanded View [file EMMM-8-1438-s009.zip › EMM_06349_EV_Source_Data/EMM_06349_source_data_FigEV3.pdf]

Figure 1 panel A

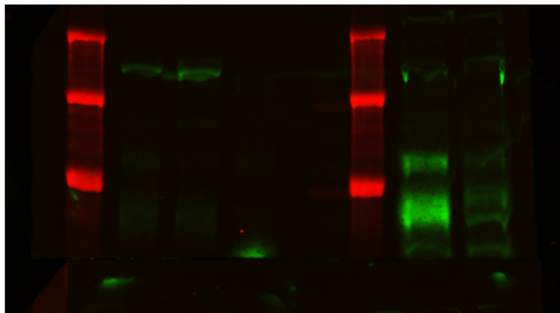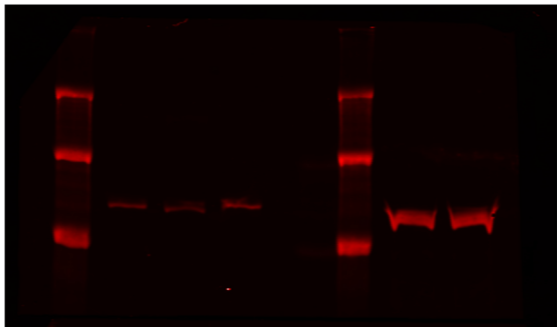

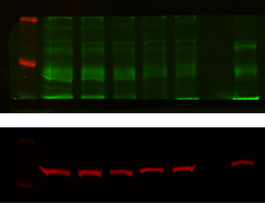

Figure 1 panel B

Figure 1 panel C

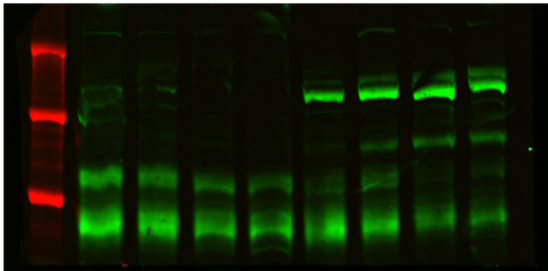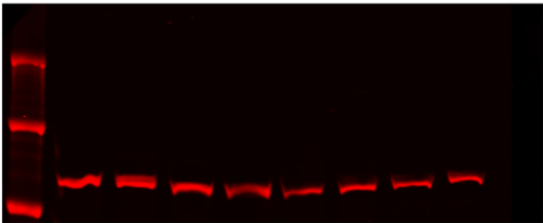

Figure 1 D

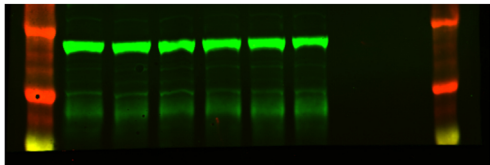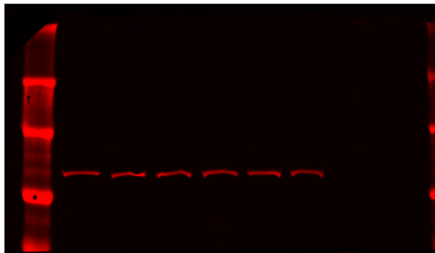

# Panel E at P2

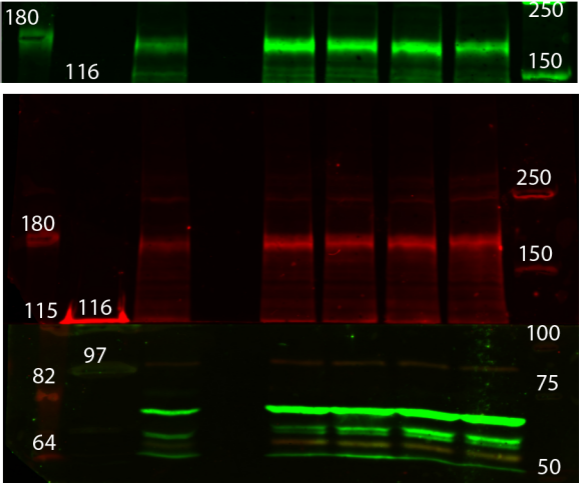

# Panel E at P10

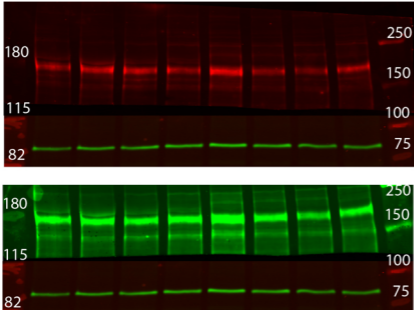

Figure 1 panel F

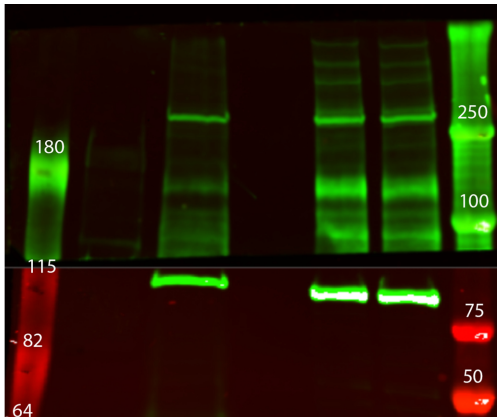

Supplement: Supplementary file 4 — Source Data for Figure 1 [file EMMM-8-1438-s002.pdf]

Figure 2 panel A

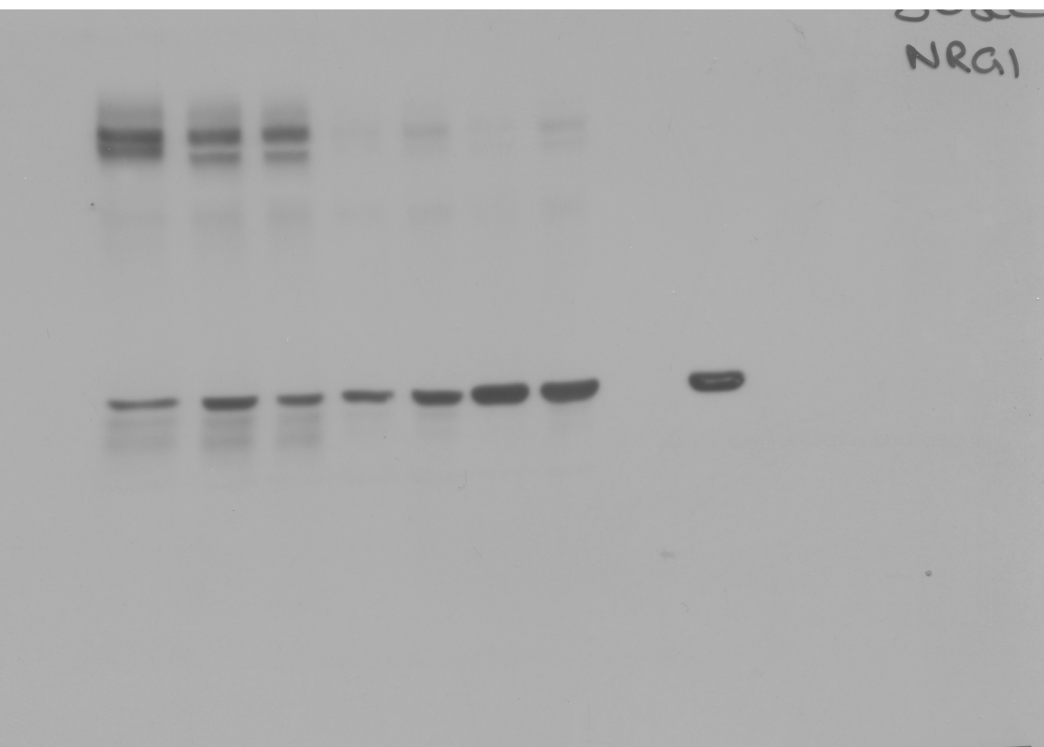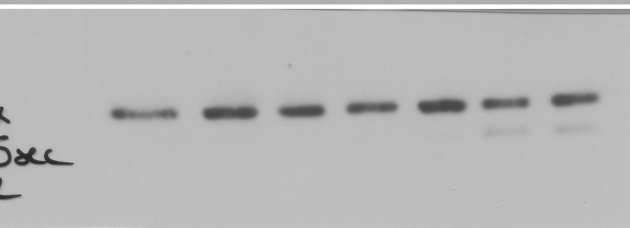

Figure 2 panel B

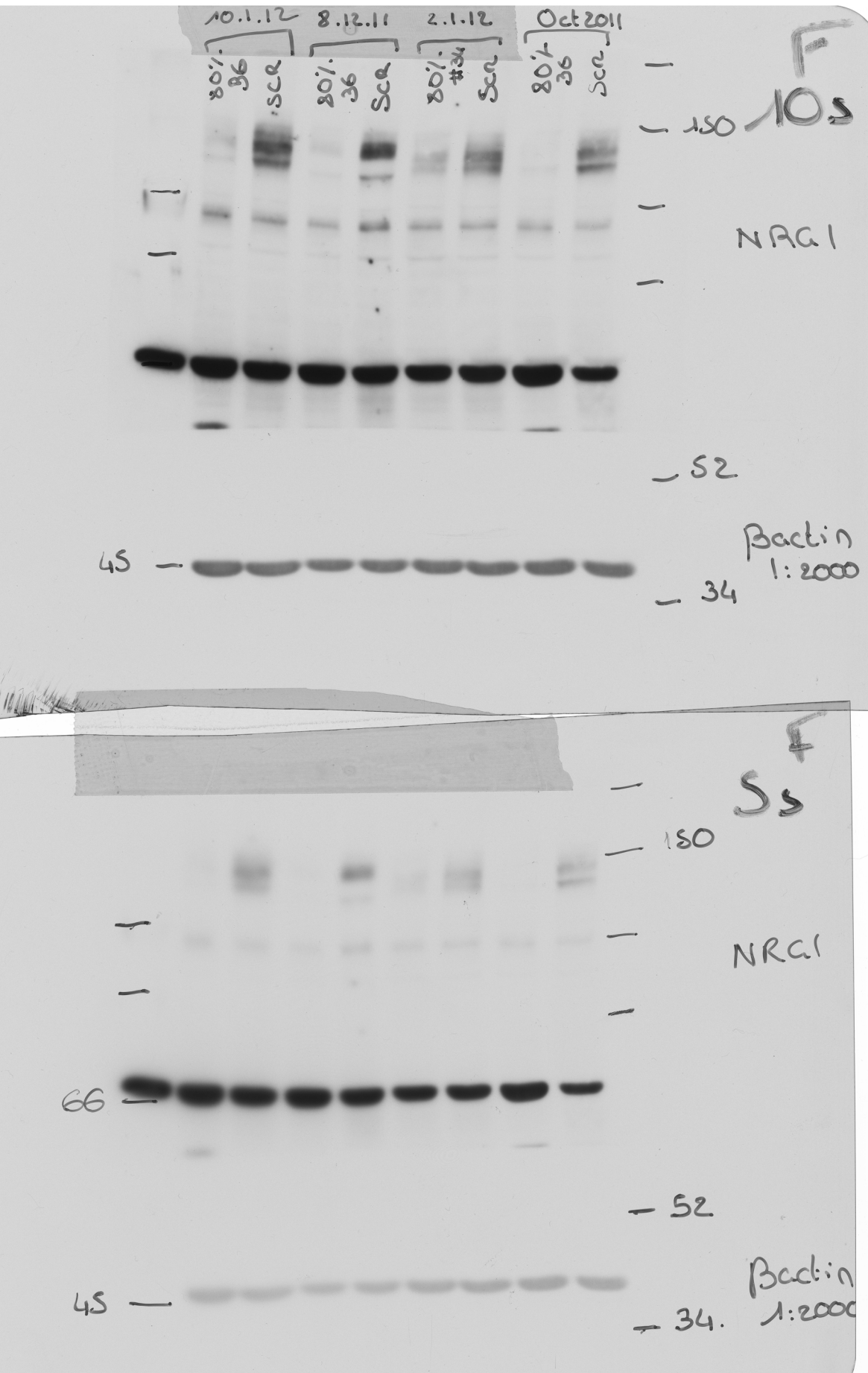

Figure 2 panel E

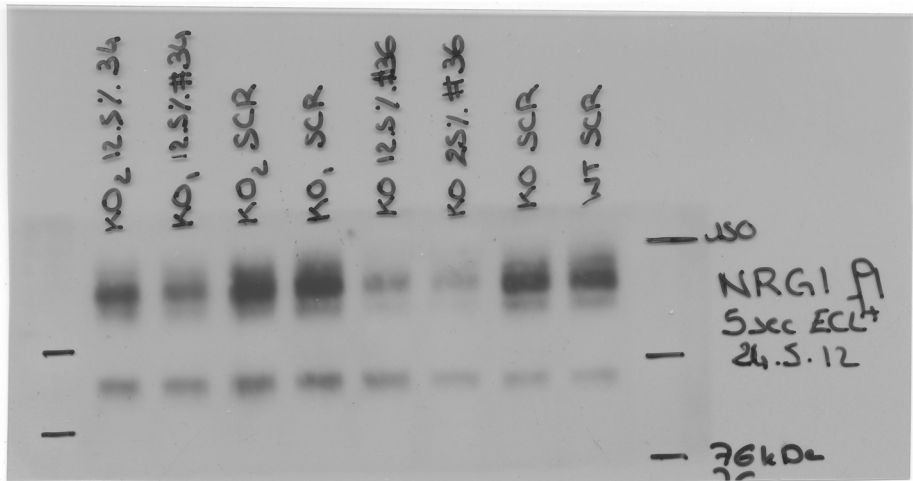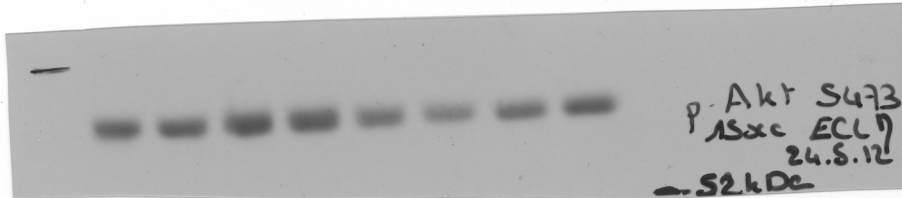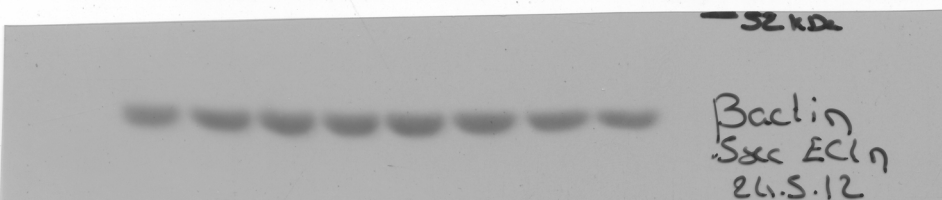

Supplement: Supplementary file 5 — Source Data for Figure 2 [file EMMM-8-1438-s003.pdf]

Figure 3 panel B

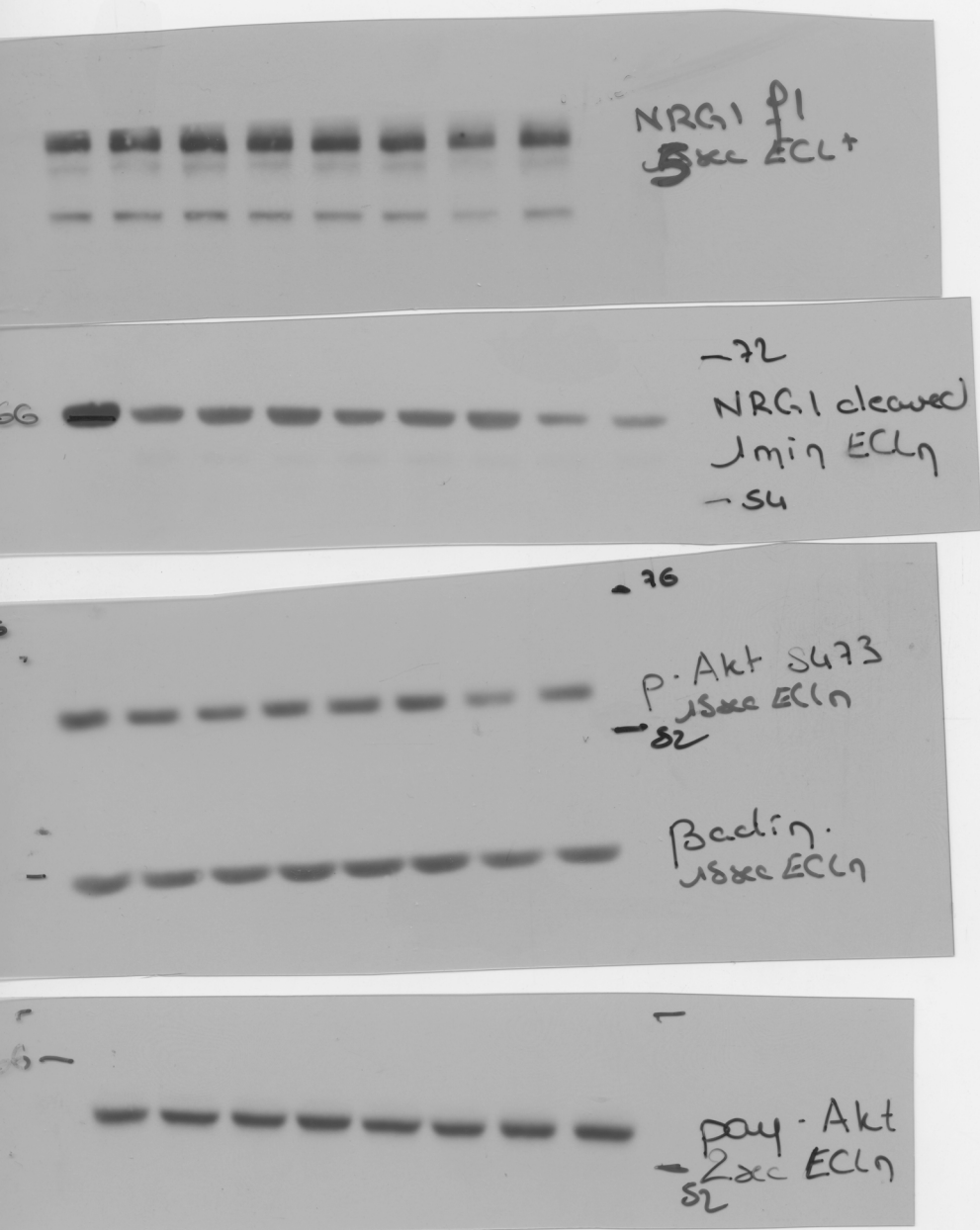

Supplement: Supplementary file 6 — Source Data for Figure 3 [file EMMM-8-1438-s004.pdf]

Figure 4 panel C

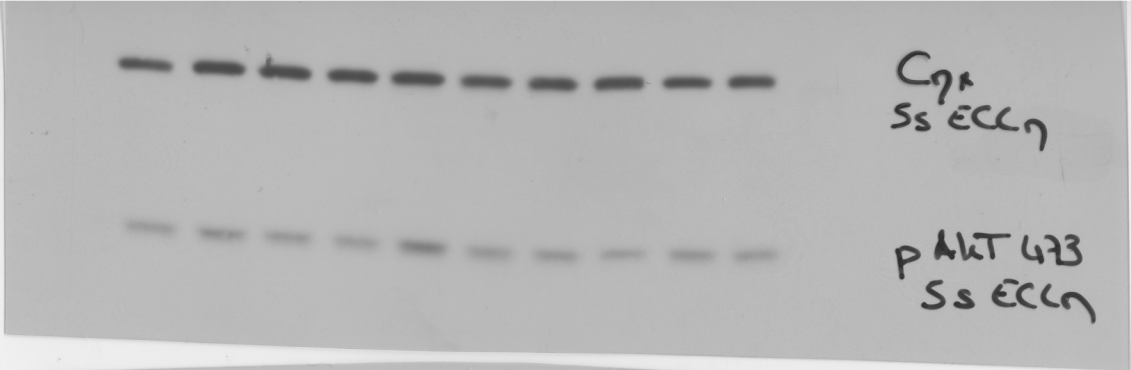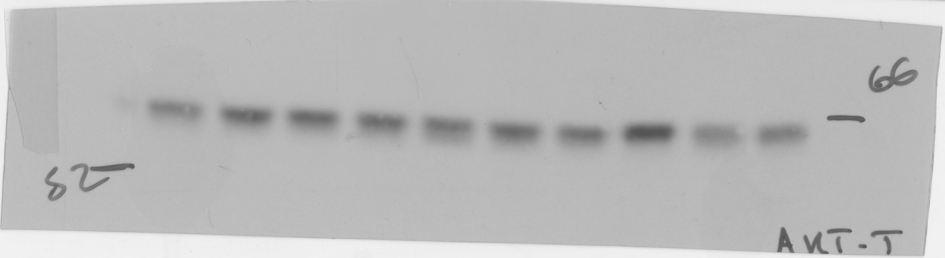

Supplement: Supplementary file 7 — Source Data for Figure 4 [file EMMM-8-1438-s005.pdf]

Figure 5 panel A

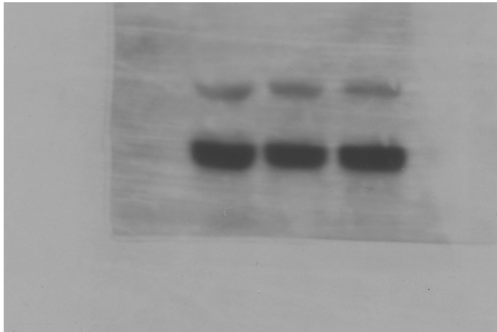

Akt tot

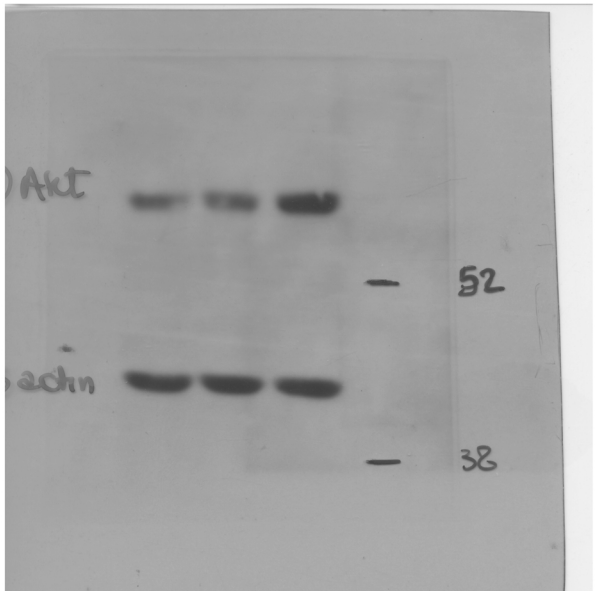

Supplement: Supplementary file 8 — Source Data for Figure 5 [file EMMM-8-1438-s006.pdf]

Figure 7 panel A

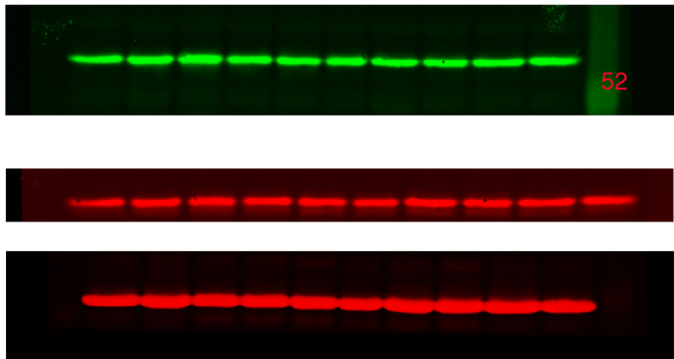

Figure 7 panel B

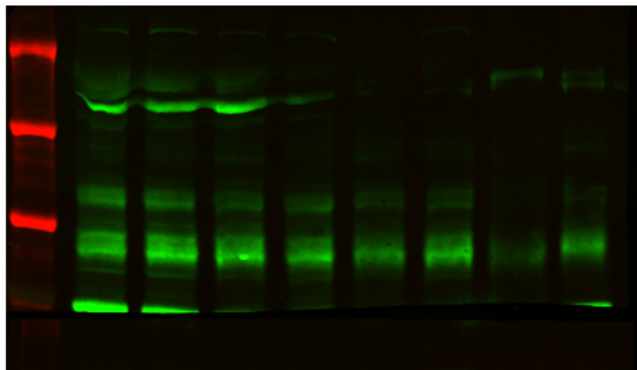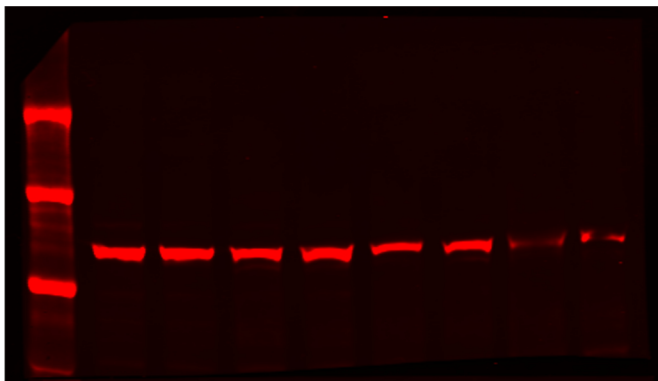

Supplement: Supplementary file 9 — Source Data for Figure 7 [file EMMM-8-1438-s007.pdf]

Figure 8 panel A

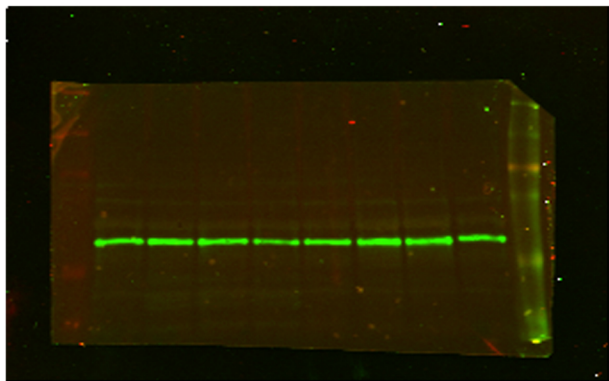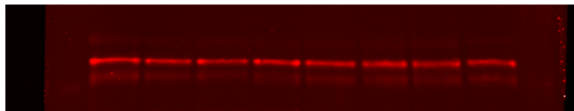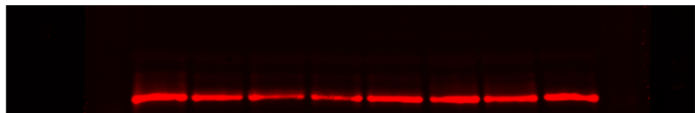

Figure 8 panel B

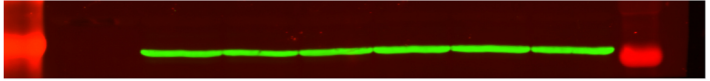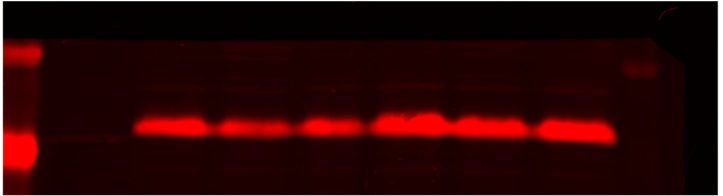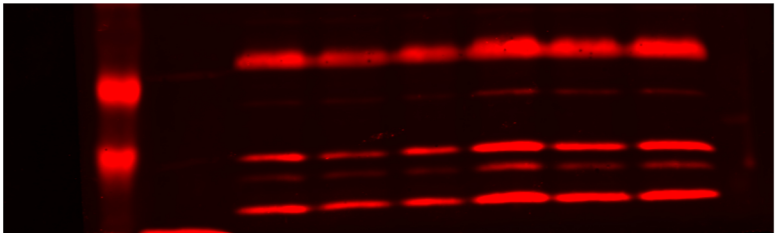

Supplement: Supplementary file 10 — Source Data for Figure 8 [file EMMM-8-1438-s008.pdf]
